# The association between social development and population health: a cross-sectional study across countries of different economic growth

**DOI:** 10.1007/s43999-022-00003-5

**Published:** 2022-06-22

**Authors:** Nikolaos Kontodimopoulos

**Affiliations:** 1 Faculty of Social Sciences, Hellenic Open University, Parodos Aristotelous 18, 26335 Patras, Greece; 2 Department of Health Economics, Medical School, National and Kapodistrian University of Athens, Mikras Asias 75, 11527 Athens, Greece

**Keywords:** Health inequities, Health policy, National income, Population health, Social determinants of health, Socioeconomic status

## Abstract

**Background:**

Social Determinants of Health (SDH) are important in explaining why some countries enjoy better health than others. This empirical study highlights controversies in the literature on the relationship between socioeconomic development and health, and investigates how the relationship might vary in countries with different economic growth.

**Methods:**

The sample consists of 172 countries, and recent cross-sectional data was collected from the World Bank’s “Data Bank”. Population health was proxied with life expectancy, infant mortality and under-five mortality, and sociooeconomic conditions were expressed with GNI/capita, unemployment rate, poverty, Gini index, educational attainment, public spending on health and physician density. Countries were categorized into three groups as low / lower middle-, upper middle- and high-income according to World Bank thresholds. Ordinary least squares regression was used to model the relationships.

**Results:**

The association between socioeconomic conditions and health differed between countries of different economic development. Poverty, educational attainment, income inequality, and physician density were the strongest contributors to health. Higher economic development had a stronger relationship with health in richer countries, and government commitment to health care in poorer ones.

**Conclusion:**

Based on evidence from studies such as this one, researchers and policymakers globally could commit to acting together on SDH, and to aligning resources from different sectors to formulate interventions aiming to improve population health.

## Introduction

The World Health Organization defines social determinants of health (SDH) as *“the conditions in which people are born, grow, work, live and age, and the wider set of forces and systems shaping the conditions of daily life”* [1]. From a SDH perspective, inequities in power, money and resources are important contributors to inequities in health, disease and mortality [2]. Much research has been conducted and documents the different ways in which social, economic, political and cultural environments affect health. The resulting evidence, although mostly descriptive, has highlighted the need for political action and interventions throughout the globe. However, data on the effectiveness of interventions aiming to modify the social determinants is scarce [3], and it is important that new studies assess such interventions and evaluate the effect of policy and practice on pathways of SDH which is a difficult task due to, among other things, the complexity of many SDH [4].

Epidemiological research consistently shows health differences among socioeconomic groups, with better outcomes usually for those in the higher strata [5]. Investigations of the relationship between economic conditions and health have revealed strong and positive correlations [6], but mostly without consensus on the underlying causal pathways. Some researchers suggest that higher income improves health outcomes [7], and others that lower income could be due to reduced productivity resulting from poor health and disabilities, and not the reverse [8]. Most research shows that higher income improves most health measures among the poor, but has a smaller effect in high incomes, both at the individual and the aggregate level. Hence, wealthier countries exhibit better population health up to a certain income level, beyond which the relationship is weak [9].

It is generally accepted that societies with unequal income distributions have worse health outcomes [10, 11]. The mechanisms by which income distribution might affect health status have been described in detail [12, 13], with compelling evidence coming from the Whitehall studies in Great Britain [14]. However, significant literature criticizes studies reporting a relationship between income inequality and health because they fail to control for factors related to income distribution and health status. For example, one study in developed countries finds no relationship between various measures of income inequality and life expectancy [15]. Another examines the relationship between income inequality and aggregate health outcomes across thirty countries over forty years and across forty-eight U.S. states over fifty years and its findings also contradict that income inequality is an important determinant of health [16].

Recent research using newer and more accurate data from more countries suggests that the effects of inequality within countries vary over time [17, 18]. Reducing income inequality of disadvantaged people will improve the health of poor individuals, help to reduce health inequalities and increase average population health [19]. Despite the lack of consensus, most recent studies have not found a strong link between the distribution of income and health, and new research with better data is critical, as income inequality has been rising in many countries [20]. However, most authors tend to agree that even if income distribution does not matter, income itself does, as do the factors with which it is correlated, e.g. wealth, educational attainment, occupation, and social class.

The association between unemployment rate and health is also debatable, with much controversy on the direction of the relationship. For example, the causality from unemployment to poor health might be explained by the limited material and financial means (i.e. poor eating or less health care spending) or the unhealthy lifestyle habits like smoking or excessive alcohol consumption, which are often linked to unemployment [21], although these harmful behaviors may vary by individual social environment [22]. Regarding poverty, and although studies are diverse in measurement approaches, methodological design, and geographical focus, there is compelling evidence that it strongly correlates with poorer health in both developing and developed countries [23]. Existing evidence on the relationship between healthcare expenditure and health is also conflicting. Some studies reveal a positive relationship [24, 25], others no significant and consistent association [26, 27], and others suggest that the effect differs between poor and rich countries [28].

Hence, the strength of the association and direction of the relationship (i.e. causality) between specific socioeconomic variables and health are unclear, and the literature is conflicting regarding both issues. This study focuses on the former, and investigates the relationship between known SDH and health proxies in groups of countries differing in income. Based on the literature, it is hypothesized that country-level indicators of population health are affected by health determinants reflecting living standards, income inequality and health system performance, and the effects vary according to the relative economic position of countries [29]. Higher economic development is expected to have a stronger association with health in richer countries [30, 31], and government commitment to health care, expressed as public expenditure on health and human health resources, to have a stronger association with health in poorer countries [32]. The study contributes empirical evidence to enhance understanding of how certain socioeconomic variables affect health in developed and developing countries, and emphasizes the importance of translating this evidence into policies to address health inequities.

## Methods

### Sample and data collection

Data was collected from the World Bank’s public “Data Bank” (https://databank.worldbank.org/source/world-development-indicators/), which is the primary compilation of international statistics on global development. Starting from 2019 and working backwards, a cross-sectional dataset was formed with mostly 2018 and 2017 data. If recent data was unavailable for some countries, the dataset was supplemented with older data (back to 2015) as these particular variables don’t change drastically from year to year. This helped to ensure that missing data would not exceed a threshold of about 10% for all variables, and that each country has one observation with data compiled from the most recent variables available. Countries meeting at least one of the following criteria were excluded from the study: i) less than 100,000 population, ii) missing data for all health variables, iii) missing data for ≥3 SDH, or iv) missing data for both public expenditure on health and physician density.

### Variable selection

The relationship between three proxy variables of population health, i.e. life expectancy (LE), infant mortality rate (IMR) and under-five mortality rate (U5MR), and known SDH such as income and income inequality, poverty, unemployment, education and health care resources is analyzed at country-level. Gross domestic product (GDP) and gross national income (GNI) are two common indices used to measure the economic scale of a country. One main difference between them is that GDP is based on location, while GNI is based on ownership, and also that GDP is the value produced within a country’s borders, whereas GNI is the value produced by all the citizens. In this study, GNI/capita was chosen to reflect each country’s national income as it correlated better with LE, IMR and U5MR than GDP/capita. According to most recent World Bank thresholds, countries are grouped by GNI per capita as: i) low-income if GNI/capita ≤ $1025, ii) as lower middle-income if GNI/capita is between $1026–$3995, iii) as upper middle-income if GNI/capita is between $3996–$12,375 and iv) as high-income if GNI/capita ≥ $12,376 [33].

The relatively small size (*N* = 27) of low-income group of countries may create multiple testing problems. To deal with this, low-income and lower middle-income countries are combined into one larger group (*N* = 74) in this study. Unemployment rate is a key indicator of an economy’s ability to generate jobs for the labor force and reflects, to some extent, the socioeconomic situation in a country [34]. However, low unemployment rate may be hiding substantial poverty, thus poverty rate defined as the proportion of the population living on less than US$5.50 per day was also used in this study. For expressing income inequality, the Gini coefficient was chosen for this study as it is the most popular in the public health literature [35]. Although young female illiteracy rate is often used as an estimate of mothers’ educational attainment and gender equality [36], this indicator is almost zero in most medium- and high-income countries, implying a skewed distribution of the variable. Therefore, educational attainment was operationalized through the number of years a child is expected to attend school. Public spending on health (% GDP) was chosen to express governmental commitment to health care as it was more strongly correlated to the health proxies than total or private spending. Physician density was used to reflect the availability of medical resources. All variables used in this study are presented in Table 1.

**Table 1:** Income groups comparisons Mean ± SD (95% CI)

	Low & lower middle income	Upper middle income	High income
Outcome Variables			
Life expectancy (years)	65.8 ± 6.0 (64.4–67.1)	73.9 ± 3.7 (72.8–75.0)	80.1 ± 3.0 (79.2–80.9)
Infant mortality /1000 births	37.5 ± 18.0 (33.3–41.6)	13.9 ± 8.4 (11.4–16.3)	4.4 ± 3.1 (3.5–5.3)
Under-5 mortality /1000	50.9 ± 27.9 (44.4–57.3)	16.4 ± 10.6 (13.4–19.5)	5.2 ± 3.5 (4.2–6.2)
Predictor Variables			
GNI/capita (1000s USD)	1.8 ± 1.1 (1.6–2.1)	7.0 ± 2.6 (6.3–7.8)	37.1 ± 19.5 (31.5–42.7)
Unemployment rate (%)	6.2 ± 5.6 (4.9–7.5)	9.6 ± 6.4 (7.8–11.5)	5.5 ± 3.3 (4.5–6.4)
Poverty rate (%)	67.0 ± 26.2 (59.5–74.4)	22.3 ± 16.1 (16.8–27.8)	1.6 ± 2.2 (0.9–2.3)
Gini index (0–100)	40.1 ± 7.6 (38.4–41.9)	41.4 ± 9.2 (38.7–44.1)	34.4 ± 6.6 (32.2–36.3)
School years (years)	9.3 ± 2.1 (8.7–9.8)	11.7 ± 1.5 (11.2–12.2)	13.3 ± 0.6 (13.1–13.5)
Public spending on health (% GDP)	2.1 ± 1.4 (1.8–2.4)	3.5 ± 1.5 (3.0–3.9)	6.0 ± 2.3 (5.3–6.7)
Physicians/1000 people	0.5 ± 0.7 (0.4–0.7)	1.8 ± 1.2 (1.5–2.2)	3.1 ± 1.2 (2.8–3.4)

### Statistical analyses

Descriptive statistics (means, SDs and 95% CIs) are reported for continuous variables. Pearson’s r measures the strength and direction of the relationship between variables. Skewed variables were logarithmically transformed to achieve a near-normal distribution. For each health indicator, multiple linear regressions were run to identify the strongest models. As different units and scales are used for the predictors (e.g. absolute numbers, dollars, percentages), standardized coefficients are reported to compare their relative importance on a common scale. Forward variable selection was used and R^2^ change, i.e. improvement in model strength when the next predictor is added, was reported. Statistical significance was set at *p* < 0.05. The OLS assumptions of normality and non-heteroscedasticity were confirmed with Shapiro-Wilk and Breusch-Pagan/Koenker tests respectively. Possible multicollinearity was examined with the variance inflation factor, which was less than the suggested threshold of 10, implying absence of severe collinearity [37]. All analyses were run with SPSS, ver. 24.0 [38].

## Results

### Descriptive statistics

By means of the exclusion criteria, 45 out of 217 countries listed in the World Bank’s databank were removed from the sample. These were mostly small islands or single-city nations with special socio-political contexts. Geographically, the excluded countries were mostly from Latin America and the Caribbean, East Asia and the Pacific region. Missing data rates for all variables, in the remaining 172 countries, were acceptable (<10%). Means and standard deviations for all variables, by country income level, were calculated. The three subgroups differed significantly both in terms of population health and socioeconomic development. Higher national income was associated with higher life expectancy, lower infant and child mortality, more school years and higher government commitment to health care. The association between country income and unemployment was unclear, as higher mean unemployment rates were observed in the two middle-income groups. Regarding income inequality (Gini index), subgroups were similar. The mean scores, for all variables, differed across the income subgroups (Table 1).

### Analysis for low- and lower middle-income countries

For the group of 74 low and lower middle-income countries, the SDH predicted 70.9% and 73.6% of the variability of IMR and U5MR respectively. Both mortality rates were predicted by the same two variables, i.e. poverty and school years, and none of the other SDHs were significant predictors in the models. For both IMR and U5MR, poverty had the strongest independent effect as shown by the standardized beta coefficients (*p* < 0.001). Regarding LE, physician density was the only significant predictor, accounting alone for 51.8% of its variability (Table 2).

**Table 2:** Regression models with the low- and lower middle-income countries (*N* = 74)

Outcome	Variables entered	Coefficients		Sig.	Model summary		
		Unstd. Beta (SE)	Std. Beta		R^2^	R^2^ change	Adjusted R^2^
Life Expectancy	Constant	69.929 (0.844)		<0.001			
	Physician density	7.572 (1.294)	0.730	<0.001	0.533	0.533	**0.518**
Infant Mortality	Constant	1.476 (0.205)		<0.001			
	Poverty	0.005 (0.001)	0.587	<0.001	0.672	0.672	0.661
	School years	−0.036 (0.015)	−0.332	0.021	0.728	0.056	**0.709**
Under-5 Mortality	Constant	1.699 (0.226)		<0.001			
	Poverty	0.006 (0.001)	0.542	<0.001	0.672	0.672	0.662
	School years	−0.050 (0.016)	−0.397	0.005	0.753	0.080	**0.736**

Only significant (*p* < 0.05) predictor variables are presented

### Analysis for upper middle-income countries

For the group of 49 upper middle-income countries, school years were an influential socioeconomic predictor of LE, IMR and U5MR, by contributing alone 54.4%, 57.4% and 57.0% explanatory power respectively to the models. It was the only significant predictor of LE, and the SDH with the strongest independent effect on IMR and U5MR according to the standardized beta coefficients (*p* < 0.001). Income inequality, expressed by means of the Gini index, contributed a further 9.2% and 8.6% explanatory power to the IMR and U5MR models respectively (Table 3).

**Table 3:** Regression models with the upper middle-income countries (*N* = 49)

Outcome	Variables entered	Coefficients		Sig.	Model summary		
		Unstd. Beta (SE)	Std. Beta		R^2^	R^2^ change	Adjusted R^2^
Life Expectancy	Constant	28.084 (7.921)		<0.001			
	School years	43.179 (7.362)	0.749	<0.001	0.560	0.560	**0.544**
Infant Mortality	Constant	3.329 (0.737)		<0.001			
	School years	−2.541 (0.598)	−0.568	<0.001	0.574	0.574	0.574
	Gini Index	0.010 (0.004)	0.358	0.013	0.666	0.092	**0.666**
Under-5 Mortality	Constant	2.596 (1.052)		0.020			
	School years	−2.795 (0.602)	−0.600	<0.001	0.585	0.585	0.570
	Gini Index	0.924 (0.354)	0.337	0.015	0.671	0.086	**0.640**

Only significant (*p* < 0.05) predictor variables are presented

### Analysis for high-income countries

For the group of 49 high-income countries, a combination of four significant SDH, i.e. poverty, public spending on health, income inequality and income per capita, in that particular order of strength according to the standardized beta coefficients, were significant predictors of IMR and U5MR, and explained 63.2% and 64.0% of the variability respectively. Public spending on health had a positive relationship with infant and child mortality, which will be addressed in the Discussion. In the LE model, income inequality and unemployment were the two significant predictors, accounting for 57.7% of its variability. In this group of high income countries, none of the health proxies were affected by educational attainment (Table 4).

**Table 4:** Regression models with the high-income countries (*N* = 49)

Outcome	Variables entered	Coefficients		Sig.	Model summary		
		Unstd. Beta (SE)	Std. Beta		R^2^	R^2^ change	Adjusted R^2^
Life Expectancy	Constant	44.135 (5.410)		<0.001			
	Gini Index	7.739 (1.130)	0.784	<0.001	0.513	0.513	0.498
	Unemployment	0.224 (0.084)	0.305	0.012	0.602	0.088	**0.577**
Infant Mortality	Constant	0.929 (0.468)		0.056			
	Poverty	0.035 (0.013)	0.429	0.012	0.508	0.508	0.493
	Public spending/GDP	0.036 (0.011)	0.441	0.002	0.571	0.063	0.544
	Gini Index	0.010 (0.004)	0.341	0.029	0.624	0.053	0.587
	GNI/capita	−0.234 (0.107)	−0.322	0.037	0.675	0.051	**0.632**
Under-5 Mortality	Constant	1.038 (0.446)		0.027			
	Poverty	0.035 (0.013)	0.438	0.010	0.523	0.523	0.509
	Public spending/GDP	0.034 (0.011)	0.429	0.003	0.578	0.055	0.552
	GNI/capita	−0.233 (0.102)	−0.332	0.030	0.628	0.050	0.592
	Gini Index	0.010 (0.004)	0.332	0.031	0.683	0.054	**0.640**

Only significant (*p* < 0.05) predictor variables are presented

## Discussion

**Fig. 1:**
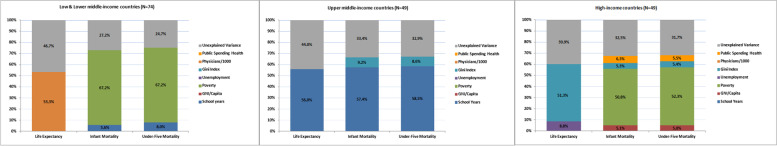
Relative contribution of SDH to population  health indicators, by country income

In this study, regression models determined the contribution of various SDH and the results are graphically summarized in Fig. 1. For each group of countries based on level of income, a proportion of the variance of each health variable remained unexplained. More specifically, for life expectancy, unexplained variance ranged from 39.9% for the high-income countries to 46.7% for the low- and lower middle-income countries. In all the groups, the variables fit the IMR and U5MR models better than the LE models, with less unexplained variance, which ranged from 27.2% to 33.4% for infant mortality, and from 24.7% to 32.9% for child mortality. In the low- and lower middle-income countries, physician density was the most important contributor to LE, whereas poverty was the dominant social determinant explaining IMR and U5MR. In the upper-middle income countries, schooling was the most dominant contributor to health in all the models, with an additional small contribution to IMR and U5MR from income inequality. For the high-income countries, income inequality the most important contributor to LE, and poverty was dominant in explaining IMR and U5MR.

The countries in this study were placed into income groups using GNI per capita as the taxonomy measure. The World Bank considers this the best indicator of economic capacity and progress and, with defined thresholds [33], four categories are formed: low-, lower middle-, upper middle- and high-income. This grouping was preferred instead of the widely accepted dichotomous taxonomy of countries as either developed or developing, as it is restrictive and more categories are often required to capture diversity in development outcomes. To avoid multiple testing problems, the World Bank taxonomy was modified here by combining low-income and lower middle-income countries into a single group. However, it should be noted that there remain unanswered questions as to the optimal number of categories and the appropriate choice of development proxy [39]. Moreover, grouping countries by economic development could widen the scope of the results in studies like this because it allows aggregating and analyzing the data for groups of similar economies, shows how different groups perform against particular social determinants, provides a useful way of tracking progress over time and facilitates a better overall understanding of social and economic outcomes. An alternative might have been to treat the countries as a single large group and adjust for social determinants (e.g. income) in the regression. However, statistical adjustment works perfectly when all the variables are measured perfectly, but in reality most variables are measured with considerable error and residual confounding is a significant problem [40].

The results of this study agree with studies having used similar predictor variables and showing a strong link between income and infant mortality [36], under-5 mortality [31] and life expectancy [41]. Additionally, our results showed a different association between socioeconomic variables and health at different country-income levels. Specifically, the association of health with income, income inequality, poverty and unemployment was significantly stronger among the rich countries, whereas the association with increased resources for health (e.g. physicians) was significantly stronger among the poorer ones. The stronger association between income and health in rich countries has also been shown in other studies which suggested that this is possibly the result of faster absorption of new medical technologies by countries which are better-off financially [30, 31, 42]. However, some studies in rich countries have not shown an association between average income and measures of health [43].

Poverty was the strongest contributor to infant and child mortality in the low- and lower-middle income, as well as in the high-income group. Two recent studies focusing on the impact of national income, inequality and poverty on population health, which used the same health variables as our study, reported similar associations between poverty and health [18, 44]. Unemployment rate was a significant predictor of LE in high-income countries, however, the sign of the coefficient was unexpected (i.e. positive association with LE). An explanation provided from a study with a similar finding is the unemployed might refrain from smoking, maintain normal body weight, be more physically active and follow a better diet [21], all of which positively affect health. In any case, the association between long-term unemployment and health has been confirmed [45, 46], but like the relationship between income and health, this relationship is also controversial with much discussion on the casual direction [22, 47].

Income inequality, expressed via the Gini index, was significant in the group of upper middle-income countries when infant or child mortality was the health proxy, and in the high-income countries in all three models. In most cases however, it was not a particularly strong predictor of these health variables, meaning that it could be affecting income through other pathways. This corroborates recent studies suggesting the absence of a significant direct relationship between inequality and health [16, 18]. In a review of 168 studies reporting the association between income distribution and population health, 78% provided statistically significant evidence, and 70% strongly supported the hypothesis that health status is worse in unequal societies, although 30% did not produce compelling evidence to support the hypothesis [48]. The implication is that if countries where wealth is spread more evenly among the population exhibit better health outcomes, narrowing the distribution might help make people healthier.

Educational attainment is important in differentiating an individual’s prospects for long life, and in this study it stood out as a very strong determinant of health in the upper middle-income countries, and also contributed to lower infant and child mortality in the low-income countries. An explanation might be that in high-income countries most people complete primary and secondary school anyway, as a result of better living conditions, and it is unlikely to affect health. On the other hand, in countries with lower income it might be attributed to many people being unable to achieve educational attainment [49]. Numerous studies have examined trends in the association between educational attainment and mortality, and despite variability in data sets and methodological approaches, all have concluded that educational differences in mortality and life expectancy have widened over the years [50]. However, some studies have suggested that higher levels of educational attainment do not necessarily extend longevity, as other factors such as parental educational attainment and income may be affecting the relationship [51].

Our results corroborate studies having shown that human health resources are positively correlated with health [24, 25, 52], as well as those that have found a stronger association in the poorer countries [31, 32]. On the other hand, we also found an unexpected positive association between public spending on health and infant/child mortality in the high-income countries, but some studies have indeed reported an inverse relationship between public spending and population health [53, 54]. More supporting evidence on the association between public spending and health in poorer countries came from a study using data from 191 countries, and showed that public expenditure is not so important in developed countries, but very much in developing economies, for improving health [55]. Studies having considered public and private health expenditure separately showed that public health spending affects longevity in a positive way, but private health spending has no impact at all [56], most likely due to private spending declining as a result of increasing public spending [57].

### Implications for policy making

This study can be seen as a contribution to understanding how socioeconomic status might affect health. However, the SDH that were shown to affect population health in poor and rich economies, are not new to the scientific community. Hence, the important thing now is to take the next step in turning the available evidence into viable and sustainable health policies addressing socioeconomic inequities and diminishing global health disparities. Despite presenting new data to enhance current knowledge this study, like many other similar ones, cannot provide “ready to use” solutions. Policymakers constantly face challenges to convert research into policy, which might extend even further if they need to process the evidence before implementing it in daily practice. An obstacle in understanding and exploiting research evidence, noted by policymakers, is the lack of proposals and/or solutions from SDH researchers. In policy formulation, offering solutions is equally important to establishing the problem, and securing policy commitment to SDH by the policymakers requires that researchers develop an awareness of the structures they aim to influence [58].

Several key health policy areas have been identified by similar studies in which policymakers are taking actions on SDH. For example, an increase in income from a refundable tax credit for low-income working families with children has been shown to reduce the incidence of low birth weight [59]. There is also evidence that pre-kindergarten improves cognitive outcomes, especially for disadvantaged children, and leads to improved educational attainment and earnings. Since children from low-income countries are less likely to be enrolled in preschool than those from higher-income countries, efforts to increase preschool access and attendance could help assure equality of opportunity [60]. Transitional jobs programs generally provide short term wage-paying work opportunities to unemployed individuals, and offer advantages to employers and employees, including positive impacts on various health outcomes such as chronic disease, mental health, domestic violence, birth outcomes, and child physical and mental health [61].

Despite these and other ongoing efforts, the translation of evidence from SDH studies into policies to reduce existing global health disparities is, undoubtedly, a complicated task and a guiding theoretical framework might be needed to explain the patterns of causality or to assess interactions between mechanisms [62]. According to Becker’s human capital theory and Grossman’s demand model, the demand for healthcare services is derived from the demand for good health which, in turn, is derived from the demand for individual utility. Furthermore, the law of diminishing marginal utility states that the more a good is consumed, the more its marginal utility diminishes. These theories could explain, for example, the documented finding that although income and education increase the marginal value of health relative to the marginal value of wealth, at higher levels of wealth and consumption like in high-income countries, only limited marginal utility is gained from additional consumption [63]. There are many similar examples showing that these concepts are important and could help policymakers in various countries to allocate the proper amount of resources to the different social groups.

Focusing even more on the SDH perspective, policymakers should aim at minimizing the accumulation of social disadvantages in order to promote better, more equitable and cost-efficient population health outcomes. Policies for disadvantaged social groups in low- and lower-middle income countries could be aimed at wealth redistribution to reduce social stratification or even the existence of social groups, or at least at eliminating unequal outcomes and consequences across these groups [64]. However, such policy changes might not be enough for health providers’ to view social interventions as equally as important to clinical ones, and financial incentives might be needed as a moderator in the process. For example, government insurers could reimburse providers who directly intervene in prioritized social determinants of health. In the US -a high income country- the Centers for Medicare & Medicaid Services is developing a pilot model that would allow healthcare organizations to bill Medicaid and Medicare for providing services such as assistance with food and housing. If successful, this could push providers to consider a person’s entire lifespan when developing their care plans [65].

### Limitations

Although recent data was used in this study, its cross-sectional design does not guarantee that this particular time “snapshot” is representative of the associations between the variables over the previous years. Future longitudinal studies could also focus on investigating business cycles, i.e. the stages in the economy as it expands and contracts (e.g. rise and fall of GDP), and their effect on the SDH-health relationship. Whenever income and health mutually determine each other, simultaneity issues usually emerge. Hence, the association might also be attributed to reverse causality, i.e. people in bad health suffer an income loss due to their inability to work [66]. In general, there is a disagreement on the direction of causality from income to health [67] or from health to income [68], and understanding the underlying causal mechanisms is crucial for improving population health, and diminishing societal health inequities. Future studies could focus on testing endogeneity bias via instrumental variables and econometric approaches such as two-stage residual inclusion. Some of the socioeconomic variables used here, e.g. poverty rate and Gini-index are estimates derived from Living Standards Measurement Surveys, which are often difficult to perform in low-income countries. Educational attainment was operationalized by the number of years a child is expected to attend school, without controlling for country context, e.g. the number of school years in developed and developing countries are probably not measuring the same thing. Finally, this study uses aggregated data, and does not address the association between individual income and individual health. It remains a question if the association between aggregate level SDHs and LE and infant/child mortality is simply due to individual level income affecting health and reflected on the aggregate level, or if there are different mechanisms between, e.g. the association between GNI and LE.

## Conclusions

The association between SDH and health varied among the poor and rich countries. Economic development had a stronger association with health in the richer countries, whereas governmental commitment to health care had a stronger influence on health among the poorer countries. Poverty also had a strong negative association with health in poorer nations, and GNI/capita was a key determinant of population health in the richer countries. Based on evidence from studies such as this, researchers and policymakers globally could commit to joining forces to act on SDH and formulate interventions to improve population health. More work is required to ensure that such commitments are carried through and that policies from different sectors align so that resources are adequately distributed to promote health throughout the globe. Finally, now might be just the right time for medical schools to prepare the next generation of physicians to consider patient’s social circumstances and to train them appropriately to address these circumstances alongside their clinical concerns.

## Data Availability

All the data for this study was collected from publically available databases.
